# Sodium Reduction in Distributive Meals Through Speed–Scratch Cooking

**DOI:** 10.5888/pcd18.210033

**Published:** 2021-08-05

**Authors:** Elinor Hansotte, Elise Gahan, Shelley Vaughn, K. Elise Lindstrom, Sandra Cummings

**Affiliations:** 1 Marion County Public Health Department, Indianapolis, Indiana

## Abstract

**Purpose and Objectives:**

Through the Centers for Disease Control and Prevention’s Sodium Reduction in Communities Program, the Marion County Public Health Department and partners implemented sodium reduction strategies in distributive meal programs (ie, low- or no-cost meals available to under-resourced populations) to meet the long-term goal of reducing the burden of sodium-related chronic disease among adults aged 60 or older. The purpose of our study was to evaluate results from the first 2 years of the program, which modified recipes to reduce overall sodium levels through speed–scratch cooking (combining prepared food products with those made from scratch).

**Intervention Approach:**

We modified recipes to reduce sodium content in 2 potato products served frequently as side dishes in distributive programs for older adults for congregate meals — those provided to groups in a community setting — and home-delivered meals.

**Evaluation Methods:**

We compared average sodium content of a 3-month menu cycle between 2 program years, the costs and consumer acceptance of recipe modifications, and consumer perceptions of product changes. Primary data included a nutrient analysis and key informant interviews.

**Results:**

Approximately 2,000 distributive meal clients of CICOA Aging and In-Home Solutions were served reduced-sodium potato dishes over the 2 years of the intervention. From year 1 to year 2, the sodium content of scalloped potatoes was reduced by 65%, and the sodium content of mashed potatoes was reduced by 87%. Client acceptance of the modified recipes met the target threshold of a mean Likert-scale score of 3.75 out of 5.0, and the combined cost savings for both potato dishes was 45 cents per serving. Key informants noted the themes of economics of cost and labor, knowledge of how to identify reduced sodium options, and quality of the replacement food as essential factors for recipe modification.

**Implications for Public Health:**

Using speed–scratch recipe modification for 2 potato dishes significantly reduced the sodium content of distributive meals for older adults. Speed–scratch recipe modification can be used as a tool to improve the nutritional value of meals and reduce the chronic disease burden of high-risk populations.

**Summary**

**What is already known on this topic?**

High sodium consumption increases risk for cardiovascular disease. Population-based interventions for sodium reduction are cost-effective and successful in reducing sodium consumption and decreasing the burden of chronic disease.

**What is added by this report?**

We used a mixed-methods approach to analyze the direct and perceived effect of 2 speed–scratch recipe modifications in meals distributed to older adults.

**What are the implications for public health practice?**

Speed–scratch recipe modification can be used to reduce sodium, save on cost and preparation time, and maintain client satisfaction.

## Introduction

*Dietary Guidelines for Americans *(1) recommends that people consume a maximum of 2,300 mg of sodium each day. The average daily American consumption of sodium is 3,400 mg (2). High sodium consumption contributes to development of hypertension. Over time, physiologic compensation for sodium-induced water retention, including increased systemic peripheral resistance and changes in endothelial function, may lead to hypertension, heart failure, and end stage renal disease (3–5). Chronic hypertension is a risk factor for cardiovascular disease because of these physiologic changes (3,6,7). According to the American Heart Association, hypertension affects 46% of Americans (8). An estimated 62% of strokes and 49% of coronary heart disorders are caused by hypertension (7). Cardiovascular disease was the underlying cause of death for 1 in every 3 US deaths in 2016 and accounted for 840,678 lives lost (8). The direct and indirect cost for treatment of cardiovascular disease in the US from 2014 through 2015 was $351.2 billion dollars (8). Hypertension and cardiovascular disease are significant burdens on the health care system (9). Multiple population-based models of sodium reduction interventions have been shown to reduce sodium consumption, lower blood pressure, and reduce the incidence of cardiovascular diseases associated with hypertension, such as myocardial infarction and stroke (10–13). Additionally, population-wide interventions have been shown to be cost-effective and often cost-saving (10,11,14).

The prevalence of hypertension and cardiovascular disease among older adults (defined as aged 65 or older) in Marion County, Indiana, is similar to or slightly greater than their overall prevalence in the same age group in Indiana and the US overall. According to a 2018 survey of Marion County residents, prevalence rates for older adults were hypertension, 69.2%; high cholesterol, 58.2%; and heart disease, 24.1% (15). In 2017, the prevalence rate for older Indiana adults was 62.8% for hypertension and 54.1% for high cholesterol. The US rate that same year was 60.5% for hypertension and 49.8% for high cholesterol (16). In 2018, the prevalence of angina or coronary heart disease for older adults was 13.4% in the US overall and 11.4% in Indiana (17).

The Sodium Reduction in Communities Program (SRCP) of the Centers for Disease Control and Prevention (CDC) works with local organizations to implement sodium reduction interventions at the population level that focus on reducing hypertension and preventing heart attacks and strokes (18). CDC awarded the Marion County Public Health Department (MCPHD) a 5-year (2016–2021) cooperative agreement to implement the program. MCPHD’s SRCP uses the following sodium reduction strategies to lower sodium intake and improve diet in distributive meal programs (ie, low-or no-cost meals for under-resourced populations): modifying meals and menus, changing procurement practices to prioritize lower-sodium foods, adhering to behavioral economics (ie, changing small factors to influence consumer choice that results in healthier eating), and implementing nutrition guidelines (18).

## Purpose and Objectives

Under the SRCP, MCPHD works with CICOA Aging and In-Home Solutions (CICOA) and their food service management company (hereinafter, food service company) Chef for Hire, to reduce sodium in their distributive meals. CICOA is Indiana’s largest Area Agency on Aging, serving more than 26% of Indiana’s older adults. In 2017, CICOA served 841,000 meals to adults aged 60 or older and adults with disabilities who participated in its Meals and More nutrition programs. Participants are provided 1 daily meal on weekdays. The program serves approximately 1,500 clients annually at neighborhood congregate meal sites (ie, meals provided to groups in a community setting) and provides home-delivered meals to approximately 1,500 additional older adults who are confined to their homes. Additionally, about 700 clients aged 60 or older participate in CICOA’s meal voucher program, through which clients can redeem vouchers for meals at participating cafeterias and restaurants. We did not include voucher meals in our evaluation, but these clients were included in CICOA-specific background data. CICOA was selected as a partner for SRCP efforts because of its previous collaborations with MCPHD and its reach in serving meals to older adults.

CICOA hosts congregate meal sites in neighborhoods with a high concentration of low-income, socially marginalized nonwhite older adults, a population with a disproportionately high burden of chronic disease. CICOA’s nutrition services play an essential role in preventing and managing chronic disease in these high-risk older adults in Marion County. A 2017 Indiana survey of older adults found that 30% of CICOA clients had heart disease, 67% had high blood pressure, and only 10% had no chronic health condition (19). The Meals and More program also fills important food and nutrition gaps by directly addressing food insecurity and nutrition. The same 2017 Indiana survey found that 47% of CICOA clients reported the cost of living in their community as “good” or “excellent,” 62% reported the availability of affordable quality food as good or excellent, 15% reported a minor problem with having enough food to eat, and 36% reported a minor problem with having enough money to meet daily expenses (19). In 2018, over 45% of Marion County adults aged 65 or older reported having some degree of worry about having enough money to buy nutritious meals (15). Adequate nutrition can help delay progression of chronic diseases and can support older adults who want to age in place at home. No additional demographic data on CICOA clients were available for our evaluation.

Indiana’s Family and Social Services Administration receives funding under the Older Americans Act for state and local agencies, including CICOA, to administer nutrition programs for adults aged 60 or older (20). The Act mandates that meals comply with *Dietary Guidelines for Americans* and also provide a minimum of one-third of daily recommended dietary reference intakes (20). The Act also authorizes states to create additional nutrition requirements and program guidance for local agencies. The Indiana Family and Social Services Administration also sets nutrition standards that meals must meet or exceed (Table 1) (21). Meeting and building upon state nutrient requirements, including sodium, can positively affect participants in CICOA’s nutrition programs.

**Table 1:** Indiana Family and Social Services Administration Nutrition Standards for Older Adult (≥65 y) Nutrition Programs

Nutrient	Requirement
Calories	A weekly average of meals that meet a minimum range of 533–733 Kcal/meal.
Total Fat	A weekly average of meals that limits total fat to no less than 20% and no more than 35% of total calories/meal.
Fiber	Must meet a weekly average of 7–10 g/meal
Calcium	A weekly average that meets a minimum of 400 mg/meal
Sodium	Weekly average must not exceed 1,000 mg/meal

An advisory team composed of project partners (CICOA and Chef for Hire administrators, chefs, dietitians, and other key personnel) and the MCPHD SRCP staff provided guidance in project planning, development, and evaluation. At the beginning of our project, the advisory team set goals to reduce sodium in CICOA distributive meals by 20% and increase the percentage of lower-sodium foods served by 20% over the 5-year project period. A subject matter expert group, a subset of the advisory team that consisted of MCPHD SRCP and CICOA registered dietitian nutritionists and a chef from Chef for Hire, held more frequent menu meetings to determine sodium reduction strategies best suited to CICOA distributive meal programs, such as menu and recipe modifications and product changes. Sodium reduction strategies considered the menu as well as chef skills necessary for modifying existing recipes and creating new speed–scratch recipes (recipes that combine ready-made, commercially prepared foods with fresh or minimally processed ingredients).

We defined lower-sodium foods as those that fell at or below the sodium guidelines the advisory team modeled after national nutrition guidelines that were lower than the Family and Social Services Administration sodium standards (Table 1). The phrase lower sodium is used because the Food and Drug Administration’s formal definition for low sodium is 140 mg of sodium or less per serving, which was not feasible for this project. Guidelines for individual food categories and the overall meal were developed so efforts could be focused on maintaining an upper limit of the meal's sodium while also providing guidance in identifying higher-sodium individual foods to be modified or replaced (Box). Our project objective was to show that sodium requirements can be met or exceeded while maintaining high client satisfaction with the food served. By using this definition of lower-sodium foods, the subject matter expert group identified high sodium meals and menu items and looked for products that could be replaced with lower sodium items and recipes that could be modified to reduce sodium content. The Advisory Team used a stealth approach to sodium reduction, which meant that clients were not informed of sodium reduction to avoid potential bias that may occur with the notion that reducing sodium compromises taste.

**Box. Marion County Public Health Department’s Sodium Reduction in Communities Program Sodium Guidelines, by Food Category**

**Table:** 

Food Category	Sodium Content (Target per Item)
Meals	800 mg
Meat and meat alternatives	480 mg
Grains	230 mg
Vegetables	230 mg
Fruit	230 mg

We evaluated the effectiveness of recipe modification as a strategy to reduce overall sodium levels through speed–scratch cooking. Secondary outcomes of interest were the cost of reduced-sodium products, preparation time for the food, and client acceptance of food modifications. Data were collected for the first project year from October 2016 through September 2017 and for the second project year, from October 2017 through September 2018.

## Intervention Approach

Recipes can be modified for sodium reduction by changing an ingredient (eg, using lower-sodium canned tomatoes), modifying the preparation or cooking process (eg, rinsing canned beans or replacing added salt with herbs), or incorporating speed–scratch methods in place of quick-prepared foods. These methods have been used successfully with customer acceptance in other settings, including senior meals programs and school meals, and by food manufacturers (22–25). In the first year of our 2-year evaluation, the subject matter expert group identified mashed potatoes and scalloped potatoes as high-sodium products that were ideal candidates for recipe modification. Because both potato dishes were frequently served as side dishes, the effect of reducing their sodium content would be greater than for a dish served only occasionally. Initially, the CICOA registered dietitian nutritionist was hesitant to change either potato dish because they were client favorites. However, the food service company was in favor of recipe modification because they wanted to improve their nutritional profile by reducing the number of meals that relied on completely processed or prepared foods. Both potato dishes were fully processed foods that could be adapted to speed–scratch preparation. 

The original mashed potatoes were made by using a fully seasoned potato pearl product that contained 330 mg of sodium per serving, and the modified version used an unseasoned potato pearl product that contained 15 mg of sodium per serving with an added lower-sodium chicken base for flavor. The potato pearls were switched from a seasoned to an unseasoned product. This change was considered a one-to-one product replacement (another sodium reduction strategy) between the original and the modified mashed potatoes. The food service company chef chose to enhance the flavor by taking the product change a step further to include the recipe modification (adding the chicken base). 

The original scalloped potatoes were made from highly processed seasoning packets and unseasoned, dehydrated potatoes. The food service company reported that opening seasoning packets was labor intensive. The modified scalloped potatoes were made with unseasoned, dehydrated potatoes to which the food service company added a reduced sodium white sauce made of shredded Swiss cheese, garlic, dehydrated onions, and a white sauce mix.

## Evaluation Methods

The MCPHD SRCP staff evaluated project activities. To thoroughly understand the impact of our recipe modifications, we used a mixed-methods evaluation approach. Quantitative data provided an understanding of the effect the changes had on the average sodium content of foods served, and qualitative data offered information on the practicality of sustaining the product replacements. Our interest was the change in average sodium content of food over 2 years of SRCP (year 1, October to December 2016; year 2, October to December 2017). The subject matter expert group used data from the first year to determine potential product replacements or recipe modifications. The 2 potato dishes were modified at the end of the first year so that the effect of the change on sodium content showed in nutrient data for the second year. We used a quantitative approach to analyze sodium and overall nutrition content of the menus for home-delivered meals and for congregate meal programs for the same 3-month period (65 meals total for each program) for both years of our evaluation. Nutrition data were collected from the food service company’s food vendor database and compiled by an MCPHD SRCP registered dietitian nutritionist. A mock data set was also created by using the sodium content from the second year’s nutrient analysis for all products except the 2 potato products, for which the sodium content from the first year’s nutrient analysis was retained.

The food service company provided cost data for procurement of specified food products. The MCPHD SRCP staff recorded both successfully trialed and implemented product replacements and recipe modifications and trialed but rejected ones on a log that included the sodium content and cost (when available) of original and replacement/modified foods and the number of meals affected by each substitution on the menus within the 3-month analysis period.

To gauge consumer acceptance of lower-sodium products (ie, foods that fell at or below the sodium guidelines as defined for our study), we conducted unmatched Likert taste test surveys on both original and modified products among congregate meal clients. The taste test surveys used emoticons to gauge food satisfaction (Figure). Foods were rated and coded for analysis on a Likert scale as 1, really do not like; 2, do not like; 3, neither like nor dislike; 4, like; and 5, really like. An SRCP evaluator, in consultation with a food service company chef and the CICOA registered dietitian­ nutritionist, set a predetermined level of 75% (Likert score of 3.75) as a goal for consumer acceptability on taste tests. We conducted taste tests for both dishes in the first year of our study. We first tested the original 2 potato dishes and then the modified potato dishes the second time those were served to clients. The desired sample size for taste tests was calculated from the total number of clients at congregate meal sites (N = 70) by using a 95% CI. Four congregate meal sites were chosen as taste test locations to assure a variety of settings, maximize the number of participants, and be feasible given staffing limitations. One to 3 MCPHD employees conducted taste tests at each congregate meal site. In addition to scoring satisfaction, we collected participant gender to compare results. The staff member(s) also sorted taste test scores by participant gender for comparison (Table 2).

**Figure:**
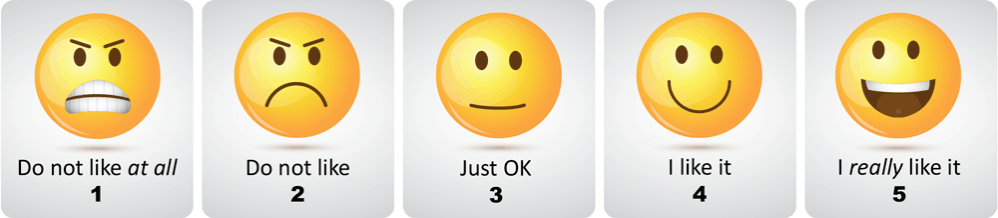
Survey tool used in Marion County Public Health Department’s Sodium Reduction in Communities Program. A predetermined level of 75% (Likert score of 3.75) was the goal for consumer acceptability on taste tests.

**Table 2:** Comparison of Taste Test Scores^a^, by Gender, for Original and Modified Potato Recipes, Marion County, Indiana, October 2016 – September 2018

Variable	Original Product Score	Modified Product Score	Change from original (95% Confidence Level)	*P *Value^b^
**Mashed potatoes**	4.30	3.92	−0.38 (−0.71 to −0.06)	.01
Women	4.17	3.85	−0.32 (−0.82 to 0.17)	.12
Men	4.43	4.00	−0.43 (−0.85 to 0)	.06
**Scalloped potatoes**	4.48	4.07	−0.42 (−0.71 to −0.13)	.01
Women	4.47	3.87	−0.61 (−1.00 to −0.22)	.00
Men	4.50	4.39	−0.11 (−0.52 to 0.30)	.62

a Foods were rated and coded for analysis on a 5-point Likert scale: 1, really do not like; 2, do not like; 3, neither like nor dislike; 4, like; and 5, really like. A predetermined level of 75% (Likert score of 3.75) was the goal for consumer acceptability on taste tests.

b Calculated by nonparametric Wilcoxon Rank-Sum test.

A qualitative approach was used to collect data on how the product changes were perceived by the food service company and CICOA. By using an interpretation of grounded theory to collect qualitative data, 3 key personnel from CICOA and their food service company were chosen to participate in key informant interviews. These participants were chosen because of their knowledge of SRCP interventions and nutrition; all 3 consented. Interviews were semistructured. Participants were sent predetermined questions ahead of time, and other probing questions based on specific interviewee responses were generated during the interviews. Interviews lasted approximately 10 minutes and were transcribed by SRCP staff members.

We used SAS Enterprise 7.1 (SAS Institute Inc) to perform statistical analyses of quantitative data. The threshold for significance was *P* ≤ .05. Data from home-delivered meals and congregate meals were aggregated to calculate the mean amount of sodium in food served in both years included in our analysis. Nutrition information was analyzed by overall meal and food category: fruit, vegetables, meat and meat alternatives, and grains. Modifications and replacements were made to products other than the 2 potato dishes in the first year. Therefore, to determine the effect that the 2 potato products had on average sodium content, a *t* test compared the actual second year sodium data with the mock data set in which the sodium content of the 2 potato dishes was held constant at the first year’s levels. The mock data set maintained all other sodium content changes made in the first year to determine the significance of the change in average sodium content of the potato dishes by food category. Descriptive analyses of categorical taste test results were completed to determine the frequency and average score of responses. A non-parametric Wilcoxon Rank-Sum test was used to determine whether the average score of the original and modified products differed significantly. We used Microsoft Excel (Microsoft Corp) to calculate the percentage of cost change by using cost data from the product replacement and modification log.

Qualitative data were coded using a thematic analysis. Two coders independently sorted transcriptions of initial codes into 6 predetermined categories: knowledge, attitudes, and beliefs about speed–scratch cooking; chef skills and knowledge; potential sustainability of modifications; client opinion; process of identifying foods for modification; and outcomes of food modifications. Together the coders broadened themes and subthemes from initial codes. Coders also highlighted compelling quotes about the practicality and sustainability of food modifications. Taste tests and key informant interviews were approved as exempt studies by the Indiana University Institutional Review Board and the MCPHD Research Review Committee.

## Results

Approximately 2,000 CICOA home-delivered meals and congregate meal clients were affected by sodium reduction interventions in the 2 years of our evaluation. Both modified potato dishes appeared on the analyzed 3-month menus multiple times, thereby increasing the effect of reduced sodium changes. In the 3-month menu cycle of the first year, scalloped potatoes were served 3 times and mashed potatoes were served 11 times at both congregate meal sites and in home-delivered meals. In the 3-month menu cycle of the second year, scalloped potatoes were served 3 times at both congregate meal sites and in home-delivered meals, and mashed potatoes were served 13 times at congregate meal sites and 12 times in home-delivered meals. Modification of the scalloped potato recipe reduced sodium by 318 mg (65%) per serving and modification of the mashed potatoes resulted in a sodium reduction of 288 mg (87%) per serving. Additionally, the modified mashed potatoes cost 32% less than the original mashed potato recipe, and the modified scalloped potatoes cost 66% less than the original scalloped potato recipe, resulting in a combined cost savings of 45 cents per serving (Table 3).

**Table 3:** Sodium and Cost Change Between Original and Modified Potato Recipes, Marion County, Indiana, October 2016 – September 2018^a^

Product	Original Sodium Per Serving, mg	Modified Sodium Per Serving, mg	Original Cost Per Serving, $	Modified Cost Per Serving, $	Change in Sodium, mg (%)	Change in Cost, $ (%)
Scalloped potatoes	490	172	0.59	0.20	−318 (−65)	–0.39 (−66)
Mashed potatoes	330	42	0.19	0.13	−288 (−87)	–0.06 (−32)

a Products were served as part of both home-delivered meals and congregate meals.

The average aggregated sodium content of meals overall for both CICOA meals (home-delivered and congregate) was reduced from 1,017 mg to 895 mg (−12%) from the first to the second year of the evaluation. Individually, both home-delivered and congregate meals had a 12% reduction in their average sodium content. Both modified potato dishes were categorized as vegetables in the nutrient analysis. Looking at vegetables only, the average sodium content for both CICOA meal types was reduced from 141 mg to 89 mg (−36%) from the first to the second year of the evaluation. Congregate meal sites had a 34% reduction in the average sodium content of vegetables, and home-delivered meals had a 39% reduction.

When comparing the actual and the mock second year data, the 2 potato dishes had a significant effect on average sodium content of the meal (average change in sodium between mock and actual data sets, −38 mg sodium/meal; 95% confidence level (CL), −60.68 to −16.05; *P* = .02) and the vegetable food categories (average change in sodium between mock and actual data sets, −73 mg sodium/vegetable; 95% CL, −133.5 to −12.05; *P* <.001). The actual average sodium content of vegetables in the second year of the evaluation was 89 mg; without modification of the 2 potato dishes, it would have been 128 mg. The actual average sodium content of meals in the second year of the evaluation was 895 mg; without the 2 potato modifications it would have been 968 mg.

Both the replacement mashed and scalloped potatoes met the 75% threshold for consumer acceptance through taste tests. Consumers did score both original potato dishes higher, and the difference was significant between the original and replacement products for both the scalloped (*P* = .01) and mashed (*P* = .01) potatoes. The qualitative key informant interviews produced multiple themes and subthemes based on the initial coding categories (knowledge, attitudes, and beliefs about speed–scratch cooking; chef skills and knowledge; potential sustainability of modifications; client opinion; process of identifying foods for modification; and outcomes of food modifications).

Because distributive meals are made in bulk and must adhere to a specific price point, the economics of cost and labor were a major theme across categories. The importance of knowing the cost and labor burden of original versus replacement products emerged in discussions of knowledge, attitudes, and beliefs about speed–scratch cooking; chef skills; sustainability of modifications; identifying modifications; and food modification outcomes. Regarding cost, 1 interviewee said, “It’s awesome when you can find the replacement that is easier for the caterer and can save cost and is a better product.” Within this theme, the scalloped potato speed–scratch modification also saved on packaging waste and labor time because fewer packages needed to be opened. To this point, 1 interviewee said, “It’s actually easier and faster to make [the modified scalloped potatoes] than the premade product.”

Another theme that crossed categories was having a knowledge base specific to identifying sodium modification in food and being familiar with substitute ingredients readily available in the vendor database or in reduced-sodium recipes. This theme appeared in categories of knowledge, attitudes, and beliefs about speed–scratch cooking; chef skills; sustainability of modifications; and identifying modifications. One interviewee said that a chef can be successful at speed–scratch cooking by “being familiar with what is out there, what kind of products you can obtain, what you can source, what you can use of food service ingredients in general.” A subtheme of both economics and the knowledge base was choosing foods for modification that appear on the menu with some frequency to make a greater impact on the desired outcomes of sodium and cost reduction.

The last major theme was the importance of producing a quality modified food product in terms of both sensory quality (appealing in taste, appearance, and texture) and nutritional quality. Interviewees reported a negligible difference in the taste, texture, and appearance between the original and modified foods. One interviewee said that the food modifications resulted in “a product that looks, cooks, and feels the same.” Related to this theme was the subtheme of client acceptance. One interviewee said that “as far as I understand, there’s been very little negative response and [the potato products] pretty much have been positively accepted” and that “probably a lot of the clients don’t even know it’s been changed.”

## Implications for Public Health

Community-level sodium reduction strategies have been proven to improve negative health outcomes associated with high dietary sodium consumption. The results from our evaluation show that a speed–scratch approach in distributive meal programs can reduce sodium, reduce food costs, and accommodate food preferences by using meal and menu modification, even for favorite recipes. Our results are similar to other studies evaluating interventions for recipe modification (or product reformulation) and sodium reduction (22,25).

Changes that lower sodium can happen quickly with collaboration and creativity, and small changes can have a substantial effect. Targeting a popular side dish can be risky, but in our study the food service company’s well-executed recipe modifications resulted in substantial sodium reduction across the many meals in which potatoes were served. Although product replacement may have been used as an intervention to find appropriate lower-sodium alternatives for these potato products, a one-to-one substitution was not always possible. Using recipe modification allowed for the food service company to maintain more indirect measures, such as food texture and appearance.

Our study had limitations. Our evaluation did not control for the experience and skill set of the food service company’s chefs. Positive outcomes associated with speed–scratch cooking as a method to reduce sodium in recipe modification in a distributive meal setting may have some reliance on chef knowledge and commitment to sustainable positive nutritional change. Future studies are necessary to know the extent to which varying levels of chef experience and knowledge influence the outcomes in similar interventions. Product replacement, rather than speed–scratch cooking as a method of recipe modification, may be a more practical starting place for sodium reduction efforts if the chef is inexperienced with this type of intervention.

Despite that limitation with respect to chefs, our evaluation does suggest that the experience, knowledge, and skills of the food service company’s chefs were a strength in our study’s setting for making sustainable product replacements and modifications that met the nutrition and cost expectations and goals of the distributive meal program. The qualitative results support chef knowledge as a key tool in this type of intervention, and the significant decrease in sodium content demonstrates the success of the speed–scratch recipe modifications in meeting project goals. Another strength of our intervention was the food service company’s willingness to collaborate with SRCP partners at CICOA and MCPHD to improve client health and nutrition.

The cost savings associated with our intervention were only realized after making the decision to use speed–scratch cooking in recipe modification, which may not be translatable across programs or food products. The only measurable cost saving in this evaluation was the direct product cost, but our qualitative analysis also indicated decreased labor time dedicated to food preparation. When feasible, future evaluations could include labor costs in their analysis.

The evaluation approach of using a 3-month menu cycle for nutrient analysis makes some assumptions about the continuity of meals throughout the year. This approach was used both to account for staffing limitations and to avoid placing additional burden on partners. CICOA maintains a relatively consistent menu throughout the year, so our approach may not be replicable for agencies with a more differentiated or seasonal approach to menu development.

The final limitation of note is that of Likert-scale surveys. The subjective nature of the response choices may lead to response bias in which respondents select only the most extreme choices. Respondents may skew toward the extreme of liking a food because distributive meal program clients are often enrolled out of need for stable meals, and they may perceive a risk of losing that service with negative feedback. The subjectivity of what it means to “like” or “not like” a food may lead to varying interpretations of what the choices mean among respondents. To address some of this potential bias, in the administration of these taste tests, we stated multiple times that responses were confidential and would not affect service.

Our detailed description of recipe modification and its effect on cost, labor, and client acceptance can help other programs seeking to reduce sodium intake make similar changes. Use of simple speed–scratch modifications can be broadly applied in sodium-reduction interventions. In our intervention, modifying dishes served many times in a menu cycle was more effective than modifying rarely served dishes. Some additional examples of incorporating speed–scratch cooking into distributive meals include adding precut, fresh tomatoes to a premade pasta sauce or salsa, adding prediced fresh bell peppers to a premade sloppy joe mix, or combining preshredded fresh rainbow carrot blend with lower-sodium apple vinaigrette to make an appealing rainbow carrot slaw. With a broader set of interventions for improving the nutritional value of distributive meals, more programs will be able to take these steps to reduce the chronic disease burden of high-risk populations.

## References

[1] US Department of Health and Human Services and US Department of Agriculture. Dietary guidelines for Americans. http://health.gov/dietaryguidelines/2015/guidelines/. Accessed April 2, 2021.

[2] Quader ZS , Zhao L , Gillespie C , Cogswell ME , Terry AL , Moshfegh A , Sodium intake among persons aged ≥2 years — United States, 2013–2014. MMWR Morb Mortal Wkly Rep 2017;66(12):324–238. 10.15585/mmwr.mm6612a3 28358799 PMC5657955

[3] Grillo A , Salvi L , Coruzzi P , Salvi P , Parati G . Sodium intake and hypertension. Nutrients 2019;11(9):E1970. 10.3390/nu11091970 31438636 PMC6770596

[4] Nijst P , Verbrugge FH , Grieten L , Dupont M , Steels P , Tang WHW , The pathophysiological role of interstitial sodium in heart failure. J Am Coll Cardiol 2015;65(4):378–88. 10.1016/j.jacc.2014.11.025 25634838

[5] Garofalo C , Borrelli S , Provenzano M , De Stefano T , Vita C , Chiodini P , Dietary salt restriction in chronic kidney disease: a meta-analysis of randomized clinical trials. Nutrients 2018;10(6):E732. 10.3390/nu10060732 29882800 PMC6024651

[6] Kong YW , Baqar S , Jerums G , Ekinci EI . Sodium and its role in cardiovascular disease — the debate continues. Front Endocrinol (Lausanne) 2016;7:164. 10.3389/fendo.2016.00164 28066329 PMC5179550

[7] McGuire S . Institute of Medicine. 2013. “Sodium intake in populations: assessment of evidence.” Washington, DC: The National Academies Press, 2013. Adv Nutr 2014;5(1):19–20. 10.3945/an.113.005033 24425717 PMC3884094

[8] Benjamin EJ , Muntner P , Alonso A , Bittencourt MS , Callaway CW , Carson AP , ; American Heart Association Council on Epidemiology and Prevention Statistics Committee and Stroke Statistics Subcommittee. Heart disease and stroke statistics–2019 update: a report from the American Heart Association. Circulation 2019;139(10):e56–528. 10.1161/CIR.0000000000000659 30700139

[9] He FJ , Pombo-Rodrigues S , Macgregor GA . Salt reduction in England from 2003 to 2011: its relationship to blood pressure, stroke and ischaemic heart disease mortality. BMJ Open 2014;4(4):e004549. 10.1136/bmjopen-2013-004549 24732242 PMC3987732

[10] Hope SF , Webster J , Trieu K , Pillay A , Ieremia M , Bell C , A systematic review of economic evaluations of population-based sodium reduction interventions. PLoS One 2017;12(3):e0173600. 10.1371/journal.pone.0173600 28355231 PMC5371286

[11] Webb M , Fahimi S , Singh GM , Khatibzadeh S , Micha R , Powles J , Cost effectiveness of a government supported policy strategy to decrease sodium intake: global analysis across 183 nations. BMJ 2017;356:i6699. 10.1136/bmj.i6699 28073749 PMC5225236

[12] Coxson PG , Cook NR , Joffres M , Hong Y , Orenstein D , Schmidt SM , Mortality benefits from US population-wide reduction in sodium consumption: projections from 3 modeling approaches. Hypertension 2013;61(3):564–70. 10.1161/HYPERTENSIONAHA.111.201293 23399718

[13] Bibbins-Domingo K , Chertow GM , Coxson PG , Moran AE , Lightwood JM , Pletcher MJ , Projected effect of dietary salt reductions on future cardiovascular disease. N Engl J Med 2010;362(7):590–9. 10.1056/NEJMoa0907355 20089957 PMC3066566

[14] Centers for Disease Control and Prevention. What is the evidence for state and local laws addressing sodium reduction among the US adult population? A policy evidence assessment report. Atlanta (GA): Centers for Disease Control and Prevention, US Department of Health and Human Services; 2019; p. 33.

[15] Marion County Public Health Department. Epidemiology Department DR 3813. Marion County 2018 Community Health Assessment Survey Report. 2019. http://marionhealth.org/2018-community-health-assessment-survey-report/. Accessed April 28, 2020.

[16] Samanic CM , Barbour KE , Liu Y , Fang J , Lu H , Schieb L , Prevalence of self-reported hypertension and antihypertensive medication use among adults – United States, 2017. MMWR Morb Mortal Wkly Rep 2020;69(14):393–8. 10.15585/mmwr.mm6914a1 32271727 PMC7147902

[17] Centers for Disease Control and Prevention. BRFSS Prevalence and Trends Data. 2019 [cited 2020 Oct 5]. https://www.cdc.gov/brfss/brfssprevalence/index.html. Accessed October 5, 2020.

[18] Centers for Disease Control and Prevention. Sodium Reduction in Communities Program (SRCP). https://www.cdc.gov/dhdsp/programs/sodium_reduction.htm. Accessed April 28, 2020.

[19] Indiana Family and Social Services Administration. 2017 CASOA - Indiana Subgroup Comparisons. https://www.in.gov/fssa/da/files/Indiana-Subgroup-Comparisons.pdf. 2017. Accessed August 10, 2020.

[20] Colello K . Older Americans Act: Nutrition Services Program. https://fas.org/sgp/crs/misc/IF10633.pdf. Congressional Research Service; 2018. Accessed September 15, 2020.

[21] FSSA Division of Aging Operations Manual. 8066 AAA policies and procedures regarding nutrition services. Indianapolis (IN): Family and Social Services Administration, Division of Aging; 2009.

[22] Riordan M , Zeitz A , Fulton B , Holliday D , Bartoli C , Aquilante J , Culinary scientists collaborating with city health department and manufacturers to improve public heath: a case from Philadelphia’s Sodium Reduction in Communities Program. J Culin Sci Technol 2020;18(6):527–34.

[23] Cox GO , Lee Y , Lee S-Y . Drivers of liking in a model retorted creamy tomato soup system with varying levels of sodium, fat, and herbs. J Food Sci 2019;84(9):2610–8. 10.1111/1750-3841.14757 31429488

[24] Losby JL , Patel D , Schuldt J , Hunt GS , Stracuzzi JC , Johnston Y . Sodium-reduction strategies for meals prepared for older adults. J Public Health Manag Pract 2014;20:S23–30. 24322812 10.1097/PHH.0b013e3182a0e3ca PMC4568554

[25] Long CR , Rowland B , Langston K , Faitak B , Sparks K , Rowe V , Reducing the intake of sodium in community settings: evaluation of year one activities in the Sodium Reduction in Communities Program, Arkansas, 2016–2017. Prev Chronic Dis 2018;15:E160. 10.5888/pcd15.180310 30576274 PMC6307830

